# The elongation factor *Elof1* is required for mammalian gastrulation

**DOI:** 10.1371/journal.pone.0219410

**Published:** 2019-07-05

**Authors:** Adam P. Tellier, Danielle Archambault, Kimberly D. Tremblay, Jesse Mager

**Affiliations:** Department of Veterinary and Animal Sciences, University of Massachusetts, Amherst, MA, United States of America; Macau University of Science and Technology, MACAO

## Abstract

Despite having been sequenced over a decade ago, the functional significance of much of the mammalian genome remains unknown. The mouse has become the preeminent mammalian model for identifying endogenous gene function *in vivo*. Here we characterize the phenotype of a loss-of function allele for the evolutionarily conserved transcription factor, Elongation Factor Homolog 1 (Elof1). Recent work utilizing the yeast homolog, Elf1, has demonstrated that Elf1 associates with the RNA polymerase II complex to promote elongation by relieving the association of the template DNA strand with bound histones. Loss of *Elof1* results in developmental delay and morphological defects during early mouse development resulting in peri-gastrulation lethality. Although Elof1 is highly conserved we observe tissue specific expression during gastrulation and in adult murine tissues, suggesting there may be other genes with similar function in diverse tissues or that mElof1 has adopted lineage specific functions. To better understand its function in mammalian transcription, we examined splice variants and find that *Elof1* regulates mutually exclusive exon use *in vivo*. Distinct from what has been demonstrated in yeast, we demonstrate that *Elof1* is essential for viability during mammalian gastrulation which may be due to a role mediating tissue specific exclusive exon use, a regulatory function unique to higher eukaryotes.

## Introduction

Although the task of evaluating the function of every gene is daunting, the International Mouse Phenotyping Consortium has made significant headway (www.mousephenotype.org). Genetically modified mice currently provide the best model to gain insight into functional role of individual genes in the mammalian genome. Approximately thirty percent of knockout genes result in embryonic lethality, which offers opportunities to study the function of essential genes during mammalian development. We are currently engaged in a large-scale evaluation of early embryonic lethal alleles generated by the Knockout Mouse Project (KOMP).

One class of proteins crucial to mammalian development and survival are transcription factors, which can control when, where, and at what rate genes are expressed in diverse tissues. Elongation factors are a subclass of transcriptional regulators that interact with RNA polymerase II (RNApII) to aid in lengthening the growing RNA chain as well as to promote proper pre-mRNA and mRNA maturation and splicing. One particularly well-conserved elongation factor, the yeast Elongation factor 1 (Elf1), was initially identified in a screen for lethality in the context of a sensitized yeast strain that was also deficient for two other components of the RNApII elongation complex: TFIIS, the first identified eukaryotic elongation factor and Spt6, a gene that encodes a histone chaperone protein and promotes chromatin-mediated elongation [1]. Despite the conservation of Elf1 from Archaea to mammals [2], Elf1 function has mainly been described in yeast and the role of the mammalian orthologue, Elof1, remains largely unknown [1, 3, 4].

Elf1 and other basal factors co-purify with the elongating RNA-polymerase II complex [4]. Recent crystal structure of the elongating RNApII complex demonstrates that Elf1 binds near the DNA entrance tunnel of the RNApII elongation complex [3]. Further cryo-EM studies have demonstrated that in cooperation with the basal elongation factors, Spt4 and -5, Elf1 promotes histone displacement in front of and behind RNApII complex to allow for elongation through the body of genes [5].

In mammals Spt6 is a well-studied elongation factor that serves to recruit several other factors to the elongation complex including Elf1, as well as Set2, a histone methylase required for the methylation of histone H3 lysine 36 (H3K36) [6–9]. Recently, many studies have shown that histone modifications influence the functional output of actively transcribed genes, including the choice and relative abundance of alternatively spliced isoforms [10–14]. Of particular interest to our findings presented herein, enrichment of H3K36 methylation has been implicated in mutually exclusive exon use [13].

These data from mammalian models combined with the co-purification data in yeast led us to hypothesize that Elof1 may regulate alternative splicing in mammalian cells. Here we present the mammalian expression pattern of *Elof1* along with, morphological and molecular characterization of homozygous *Elof1* knockout embryos during early development. Unlike yeast, we find that a homozygous loss of function Elof1 allele is lethal in mammals and our data suggest that the failure during early development is due, in part to aberrant alternative splicing in *Elof1* mutant embryos.

## Materials and methods

### Embryo production and genotyping

Timing of embryonic development was determined by presence of a vaginal plug the morning after mating (E0.5). Embryos for analysis were generated by mating male and female mice heterozygous for an *Elof1* knockout allele, *B6N(Cg)-Elof1*^*<tm1*.*1(KOMP)Vlcg>/J*^. Genotype of each embryo was determined by PCR using primers to amplify the wildtype and mutant alleles (Wildtype: 5’ CTTTCTGCAACCACGAGAAG’ and 5’TGACTCCAGTGTTTCGAGCA3’; Mutant: 5’GCCTCCATCCCTACAGTCAC3’ and 5’ CGGTCGCTACCATTACCAGT3’). During gastrulation, genotypes were recovered at expected Mendelian ratios. All mice used were maintained on the C57BL/6NJ background. All animal experiments were approved by UMASS IACUC.

### RNA-extraction and cDNA synthesis

RNA extraction was performed with Roche High Pure RNA Isolation Kit (Roche 11828665001). cDNA was synthesized with both random hexamers and oligo-dT primers as described previously [15]. RT-PCR was performed with 1ul template for 35 cycles of 30 seconds at 60C, 72C and 95C with the following gene specific primer pairs (5’ to 3’): *ActB* (GGCCCAGAGCAAGAGAGGTATCC and ACGCACGATTTCCCTCTCAGC); *Elof1* (GGACGAAGAAAGTCCAAACG and GGCATCTATCCAGTCGCTGT); *Fgfr2* constant (CAGCGAGAAGATGGAGAAGC and GTCTGACGGGACCACACTTT); *Fgfr2-IIIB* (CCAAGAAGCCAGACTTCA and AGGCAGACTGGTTGGCCTG); *Fgfr2-IIIC* (CCAAGAAGCCAGACTTCA and CAACCATGCAGAGTGAAAGG). Relative quantification was performed using GeneTools® software. For splice variants, the relative abundance was calculated using the quantification of the “constant” amplicon as the denominator and -IIIB or IIIC as the numerator to calculate the relative isoform abundance. A student’s T-test was used to determine significance.

### Fixation, embedding, and sectioning

Embryos were prepared for histology by fixation in 4% paraformaldehyde (PFA) overnight at 4C. Embryos were dehydrated through a series of ethanol washes; 20 minutes each in 25%, 50%, 75% ethanol diluted in phosphate buffered saline/0.01% tween20 (PBT), followed by two 100% ethanol washes. Embryos were embedded and sectioned as previously described [15].

### Immunofluorescence

Sections were de-paraffinized with three 10-minute xylene washes and rehydrated with three 5-minute washes in 100% ethanol, followed by successive 1-minute washes in 90%, 80%, 70% ethanol and water. Antigen retrieval was performed by boiling for 5 minutes in 0.01M Citric Acid Buffer pH = 6.0. After slides cooled to room temperature they were washed twice in PBT for 2 minutes and blocked with 0.5% milk in PBT for 2 hours at room temperature in a humidified chamber. Primary antibody was applied in 0.05% milk/PBT overnight at 4C in a humid chamber. Three 15-minute PBT washes preceded a 1-hour secondary treatment in 0.05% milk/PBT in a humid chamber at room temperature. Slides were washed in PBT for 15 minutes twice and then in PBS for fifteen minutes. Slides were then stained with 4′,6-diamidino-2-phenylindole (DAPI, Roche or Molecular Probes) in PBS (1:10,000) for 2 minutes and then rinsed with PBS. Slides were sealed and coverslipped with Prolong Gold (Invitrogen P36934). Primary antibodies were used at the following concentrations: Oct4 [Santa Cruz, sc-8629 (1:500)], E-cadherin [BD Biosciences, 610181 (1:500)], PH3 [Abcam, ab5176 (1:500)], Brachury [Santa Cruz, sc-17743 (1:250)], Caspase3 [Abcam, ab13847 (1:250)]. Secondary antibodies were diluted 1:500 and included Alexa Fluor 488 donkey-anti-goat [Molecular Probes (A-21206)], Alexa Fluor 546 donkey-anti-mouse [Molecular Probes (A-11056)] and Alexa Fluor 546 donkey-anti-rabbit [Molecular Probes (A-11056)].

### Hematoxylin & eosin staining

Following immunofluorescence, cover slips were removed in PBS and then washed in Tap water for 1 minute. Slides were then stained with hematoxylin for 3 minutes and then placed under gently running tap water for 1 minute. Slides were then dipped into 0.3% acid alcohol, placed under gentle running tap water again for 1 minute, submerged in Scott’s Tap Water Substitute (20 h MgSO_4_, 3.5g NaHCO_2_/L dH_2_O) for 1 minute and then washed in still tap water for 1 minute. The slides were then placed into 95% ethanol for 1 minute, stained with eosin for 3 minutes and then again washed in 95% ethanol with two successive 1 minute washes in 100% ethanol. Lastly, the slides were washed with xylenes three times for 1 minute each.

### X-gal (5-bromo-4-chloro-3-indolyl-β-D-galactopyranoside) staining

Freshly dissected embryos were fixed in X-gal buffer containing 0.2% glutaraldehyde and 1% formaldehyde on ice for 15 min and subjected to modified protocol from Tremblay 2000 [16].In brief, the fixed embryos were washed with X-gal buffer (PBS, 5 mM EGTA, 2 mM MgCl:6H2O, 0.2% NP-40, 0.2 mM deoxycholate) for 10 minutes three times, and stained with X-gal stain (X-gal buffer, 5mM potassium ferricyanide and 5mM potassium ferrocyanide and 0.5 mg/ml X-gal) overnight at 37°C. Subsequently, embryos were dehydrated in ethanol, cleared in xylene, embedded with paraffin, and sectioned at 7μm thick. The sectioned embryos were deparaffinated and rehydrated for subsequent processing. Eosin staining was performed by immersing rehydrated sectioned embryos in eosin Y solution for 15–20 seconds, followed by 95% ethanol for 2 minutes and then 100% ethanol for 2 minutes, and lastly cleared in xylene. Slides were then sealed with Cytoseal 60. X-gal stained sections were imaged with a Panoramic MIDI II slide scanner (3dHistech).

### Imaging

Digital images of whole mount embryos were captured on a Nikon SMZ-1500 stereomicroscope equipped with a Spot Idea Digital Camera and Spot software (v4.6). Digital images of sectioned embryos were taken with a Nikon Eclipse TE2000-S inverted fluorescence microscope and QImaging Retiga Exi Fast 1394 camera and NIS-Elements BR Software or were captured using a Perkin-Elmer Midi slide scanner.

## Results

### Defective gastrulation in *Elof1* mutant embryos

Prior to gastrulation at embryonic day 6.0 (E6.0), the embryonic portion of the mammalian conceptus is comprised entirely of the embryonic ectoderm–a single epithelial layer of cells in the radially symmetric “egg cylinder” termed the epiblast. At E6.5 the cup shaped epiblast initiates a delamination process via an epithelial to mesenchymal transition that converts the epiblast to mesoderm and endoderm layers. This process initiates from the primitive streak, a specific group of cells that morphologically define the posterior portion of the embryo. The primitive streak initiates at the embryonic/extraembryonic junction on the posterior side of the embryo and extends to the distal portion of the embryo by E7.5. After streak elongation, the embryonic organizer, termed the node, forms at the anterior end of the streak. This structure represents one of the morphological and molecularly identifiable hallmarks of a typical E7.5 embryo. Other representative structures include the notochord, an axial midline structure that extends from the node to the prechordal plate at the anterior end of the embryo, headfolds which are produced by the anterior ectoderm and the embryonic mesoderm-derived allantois that projects from the posterior end of the primitive streak into the extra-embryonic cavity.

**Fig 1:**
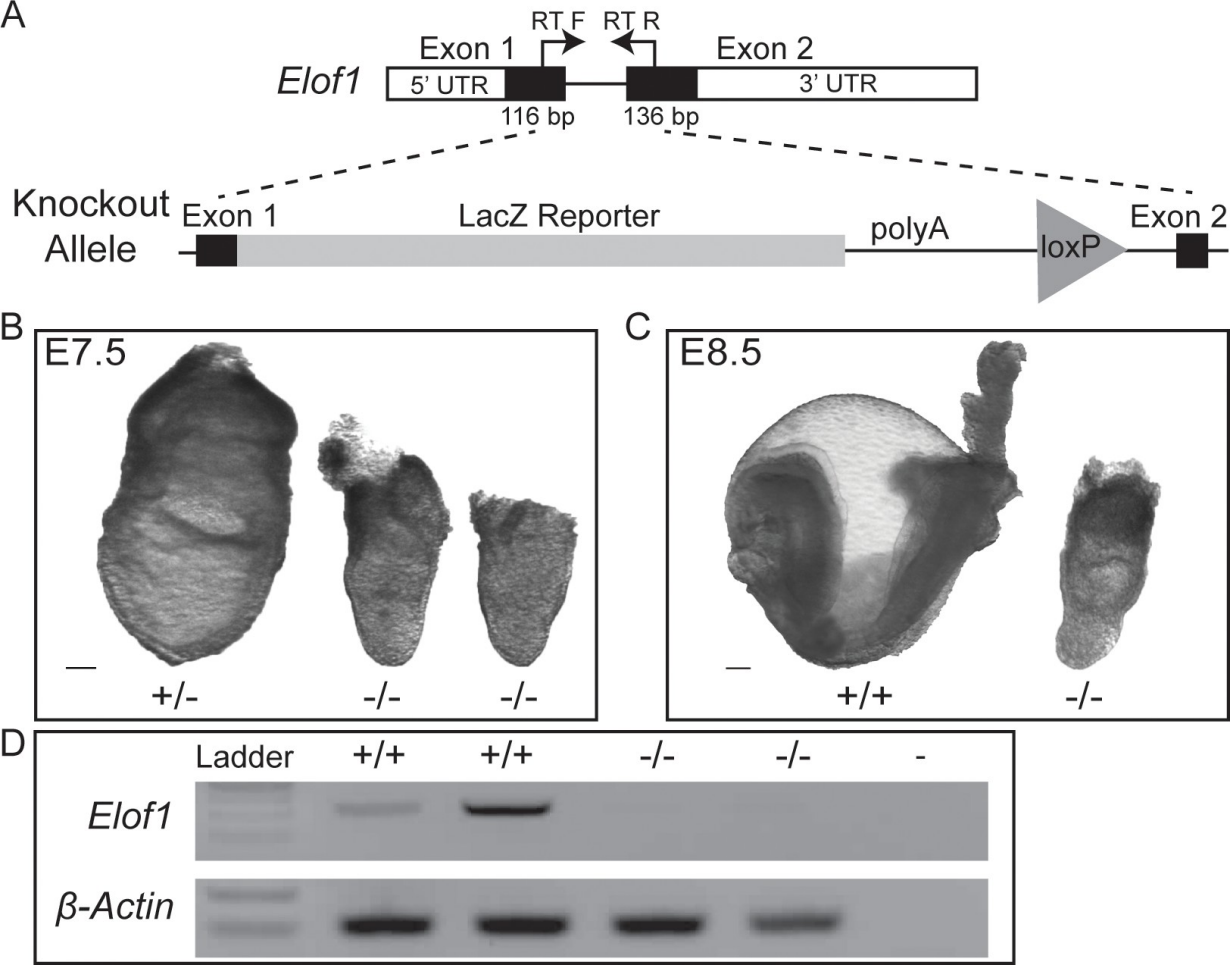
Elof1 knockout embryos show severe morphological defects. A) A schematic of the wildtype (upper) and the mutant *Elof1*^*<tm1*.*1(KOMP)Vlcg>/J*^ allele (lower) indicating the deleted endogenous sequence, the knock-in reporter construct and the primers used for RT-PCR. B.) Heterozygous littermate (left) and 2 mutant embryos at E7.5. C) A heterozygous littermate and a mutant dissected at E8.5. D) RT-PCR analysis reveals no detectable *Elof1* mRNA in homozygous mutant embryos. Scale bars equal 100uM. “-/-” denotes homozygous mutant embryos; “-” refers to no template control.

The Elof1 allele utilized herein (B6N(Cg)-*Elof1*^<tm1.1(KOMP)Vlcg>/J^) was generated by the Jackson Laboratory as part of the KOMP. This allele replaces a portion of exons 1 and 2 with a lacZ reporter (Fig 1A). As reported by the IMPC, heterozygous animals are viable and fertile, but display hypoactivity, increased circulating sodium and decreased circulating glucose (www.mousephenotype.org). Heterozygous intercrosses failed to produce any homozygous B6N(Cg)-*Elof1*^<tm1.1(KOMP)Vlcg>/J^ pups nor any homozygous embryos (hereafter referred to as “mutant allele” or “mutant embryos”) at E18.5, 12.5 or 9.5, demonstrating that this genotype is early embryonic lethal [17, 18].

To initiate our analysis, we crossed Elof1 heterozygous animals and dissected litters at E7.5 and E8.5 (Fig 1B and 1C). All embryos were genotyped and homozygous mutant embryos were recovered at expected Mendelian ratios (28% homozygous mutant (n = 18), 48% heterozygous (n = 31), 24% homozygous wildtype (n = 16)). Although there is sublte size variation among mutant embryos, all mutants display obvious morphological defects resulting in developmental failure (described below). To ensure that *Elof1* transcription was altered as expected, we performed RT-PCR from RNA extracted from control and mutant littermates. Using intron-spanning primers designed to amplify the wild-type transcript (RTF + RTR in Fig 1A), we confirmed that all embryos with the abnormal phenotype also lacked the endogenous *Elof1* transcript (Fig 1D).

### *Elof1* is differentially expressed in embryonic and adult tissues

**Fig 2:**
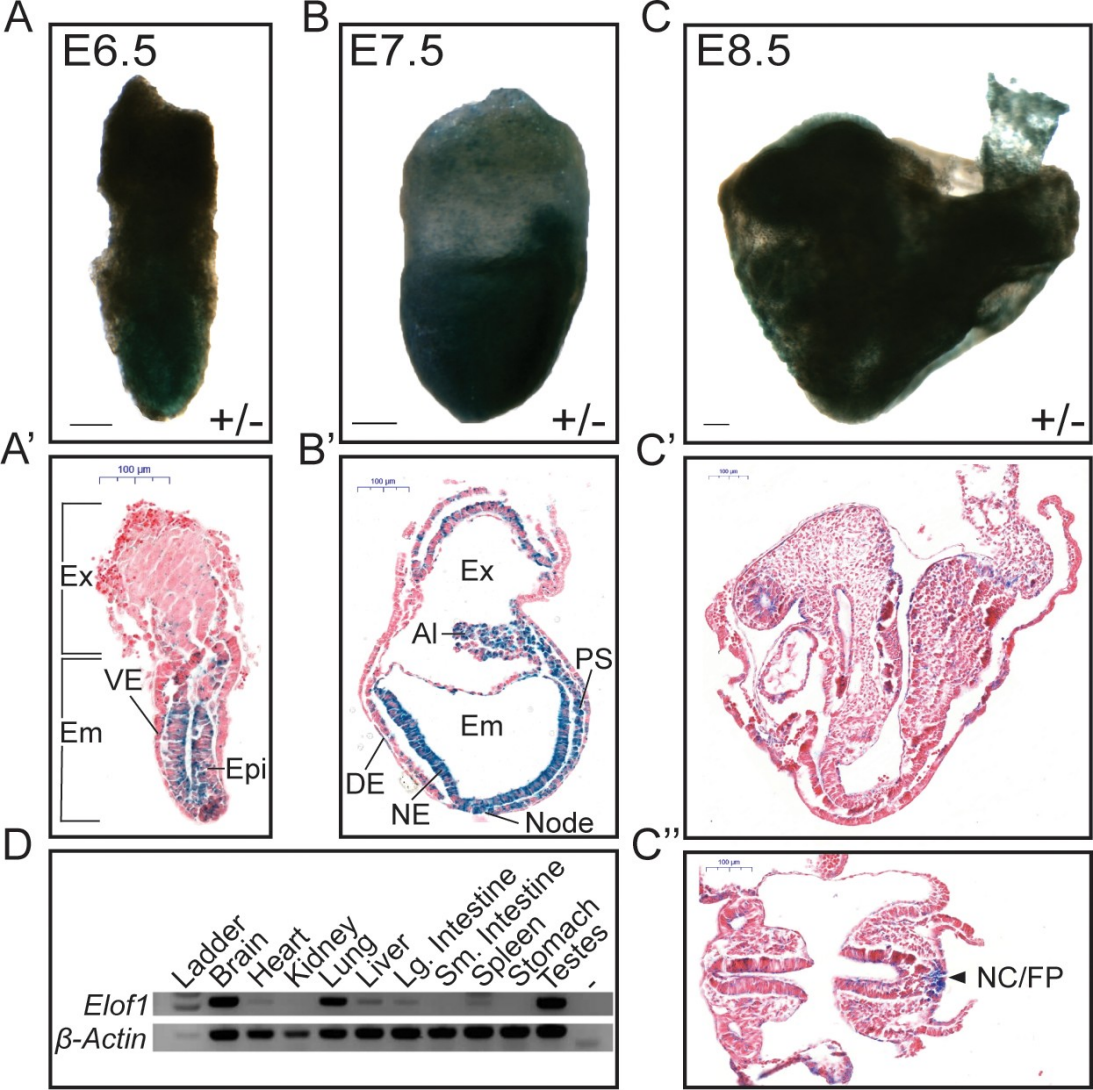
*Elof1* is differentially expressed in embryonic tissues. A-C) Whole mount (A-C) and corresponding sagittal sections (A'-C') of E6.5, 7.5 and 8.5 X-Gal stained heterozygous embryos. C" is a transverse section of a representative E8.5 X-Gal stained embryo. Scale bars are 100μm. D) RT-PCR for *Elof1* in a variety of heterozygous adult tissues. “-” is the no template control.

To begin to assess the early defects caused by loss of Elof1, we used the *lacZ*-reporter to examine *Elof1* expression in heterozygous embryos. At E6.5, lacZ-expression is strongest in the epiblast (Epi) with a notable lack of signal in the visceral endoderm (VE), a non-primitive streak derived extra-embryonic tissue that will produce a portion of the yolk sac (Fig 2A). Some visceral and parietal endoderm cells had extremely weak X-gal puncta, which are likely remnants from expression at earlier stages. Similarly, at E7.5 the *lacZ* reporter is highly expressed in all epiblast derivatives including the primitive streak (PS) derived mesoderm (both embryonic and extra-embryonic mesoderm), the node, neurectoderm (NE), primitive streak and definitive endoderm (DE, Fig 2B). Intriguingly, by E8.5 the majority of tissues show no or weak *lacZ* expression, with notable expression in the notochord and floorplate of the posterior neural tube which maintain robust *lacZ* expression (arrowhead, Fig 2C”).

To confirm that the lacZ-reporter correlates with endogenous *Elof1* expression we performed both RT-PCR and lacZ staining on a variety of adult tissues collected from heterozygous animals (Fig 2D). We observed robust *Elof1* mRNA in brain, lung and testes, and a lower level of expression in heart, liver, large intestine and spleen. *Elof1* mRNA was absent in kidney, small intestine and stomach. Importantly, these RT-PCR results are consistent with wholemount lacZ staining performed by the IMPC (www.mousephenotype.org/data/genes/MGI:1913376#expression) with heterozygous adult tissues, indicating that the lacZ reporter recapitulates endogenous *Elof1* expression.

### *Elof1* mutants fail to initiate gastrulation

**Fig 3:**
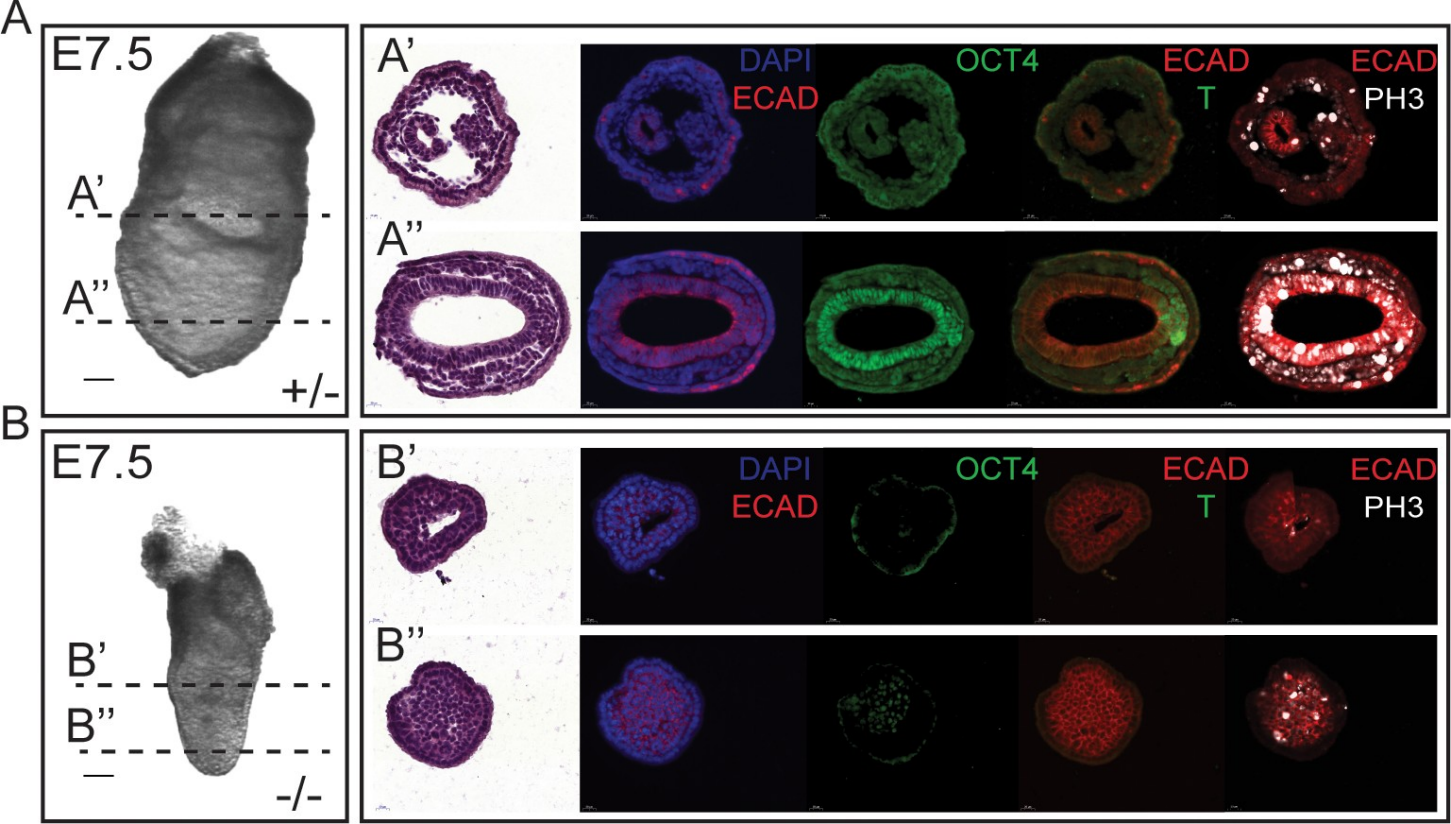
*Elof1* mutant embryos fail to gastrulate. A-B) Whole mount images of an E7.5 control (A) and mutant (B) embryo. Transverse sections were performed as indicated on the intact control and mutant (A', A" and B', B", respectively) embryo. The section in each row was subject to H & E staining to reveal morphology, DAPI to reveal nuclei and also subjected to immunofluorescence with antibodies recognizing E-Cadherin, Oct4, T and PH3 as indicated. Scale bars indicate 50μm.

At E7.5, mutant embryos resemble E6.5 wild-type in overall size and shape, and many mutants have an abnormal buildup of cells in the embryonic portion of the embryo (Fig 1B, mutant on left and Fig 3B), while others have a severe reduction of the proximal-distal length of the embryo proper (Fig 1B, mutant on right).

Given the developmental delay and lack of recognizable structures at E7.5, it is not surprising that these defects are more pronounced in E8.5 mutant embryos. By E8.5, the mutants have increased in size (relative to E7.5 mutants) but remain considerably smaller than littermates and lack all structures typical of E8.5 embryos, including the absence of obvious headfolds, heart, somites or allantois. Importantly, even at E8.5 the features typical of gastrulating embryos at E7.5 (primitive streak, node, and early headfolds) are not observed in mutants, indicating that while the overall size of mutants increase, developmental progress does not. Despite the lack of embryological hallmarks, most mutants possess a clear extra-embryonic/embryonic boundary indicating that pre-gastrulation events leading to egg cylinder formation had occurred properly. The mutant extra-embryonic tissues possess an appropriately sized exocoelemic cavity and a clear distinction between the visceral endoderm and extra-embryonic ectoderm, further suggesting that these two extra-embryonic lineages are less perturbed than the embryo proper.

To further explore the structural and molecular defects that occur in the absence of Elof1, control and mutant embryos were paraffin embedded, sectioned and subjected to immunofluorescence (IF). To be able to directly compare our IF results with histological information we developed protocols to perform two rounds of multi-color IF followed by hematoxylin and eosin (H&E) staining on the same slide (Fig 3). These assays reveal that both the embryonic and extra-embryonic compartments of mutant embryos are filled with small and abnormally rounded cells These abnormal cell are neither cuboidal nor mesenchymal in shape, as would be expected for epithelial and mesodermal tissues, respectively, observed in control embryos (compare 3A’-A” to 3B’-B”). These analyses suggest that neither the primitive streak nor any streak derivatives form properly and that the epiblast cells instead adopt a uniform atypical shape in the absence of *Elof1*. At E7.5, the internal-most layer of ectoderm is normally columnar (Fig 3A’ and 3A”), however in mutant embryos all interior cells are rounded suggesting that the epiblast has either morphologically transformed or died (Fig 3B’ and 3B”).

The normal morphogenetic events that occur during gastrulation are accompanied by well-documented lineage-specific gene expression. Oct4 is a pluripotency and germ cell marker that is expressed in the inner cell mass (ICM) at E3.5 and in the ICM-derived cells of the E4.5–6.5 epiblast in wild-type embryos. As cells of the epiblast differentiate during gastrulation, Oct4 is silenced. In the E7.5 embryo, Oct4 marks the uncommitted epiblast, newly formed primitive streak cells and the primordial germ cells located at the posterior junction of the embryonic/extra-embryonic boundary (Fig 3A’ and 3A”). As cells ingress through the primitive streak and assume a mesodermal fate they express the nascent mesodermal marker Brachyury (T).

To better assess the mutant embryos, we used immunofluorescence to examine the expression of Oct4, T, the epithelial marker E-Cadherin, and the proliferation marker phospho-histoneH3 (PH3). In the E7.5 mutant embryos there are no obvious Oct4 positive cells in the epiblast except for a subset of the distally located rounded cells mentioned above (Oct4, Fig 3B’ and 3B”). Because epiblast cells are normally highly proliferative, we also assessed PH3 as a proxy for cell division. Although PH3 positive cells are abundant in control embryos (PH3, Fig 3A’ and 3A”) mutants contain far fewer (PH3, Fig 3B’ and 3B”). Interestingly, the only mutant cells that were PH3 positive were those that were also Oct4 positive (PH3,Fig 3B’ and 3B”). Although T expression was abundant in the streak and nascent mesoderm of controls, T was absent in mutants underscoring the fact that the primitive streak had failed to initiate. Combined, these results demonstrate that E7.5 mutant embryos fail to initiate gastrulation.

### Elof1 regulates alternatively spliced mRNA variants

Genome-wide investigations of the distribution of histones, histone variants and histone modifications have revealed that they are deposited in specific patterns at many coding sequences in mammalian genes. Several studies have shown correlations between specific histone modifications and the regulation of alternative splicing [13]. Components of the RNApII elongation complex interact with histones and this complex also plays important roles in pre-mRNA splicing (reviewed in [19]). The yeast orthologue of Elof1, Elf1, interacts with Iws1 (Spt1) to facilitate histone H3K36 trimethylation (H3K36me3) modification, and recent studies show that Iws1 is also essential for early murine development [20, 21]. In higher eukaryotes H3K36me3 is linked to alternative splicing through the recruitment of polypyrimidine tract-binding protein (PTB) [22, 23]. PTB is an RNA splicing factor that binds to and represses the use of specific exons [24]. One well studied PTB-dependent alternative splice variant is Fibroblast Growth Factor Receptor 2 (FGFR2). FGFR2 has two known isoforms that differ at their third IgG like receptor loop. These two isoforms are FGFR2- IIIb and -IIIc, which each bind different FGFs to promote distinct cellular responses [13, 25–27]. Because FGFs play diverse roles in development (reviewed in [28, 29]), including documented functions during gastrulation, we hypothesized that altered splice variants may play a role in the defects observed in *Elof1* mutants.

**Fig 4:**
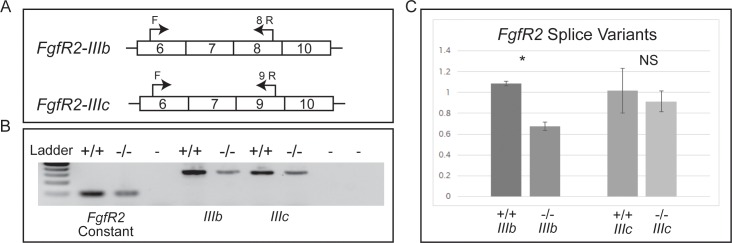
*Elof1* alters PTB-dependent alternative splicing. A) Schematic representation -*IIIb* and -*IIIC* transcripts produced at the *Fgfr2* locus. B) RT-PCR with primers specific for a constant region of *Fgfr2* mRNA as well as *-IIIb* and *-IIIc* specific amplicons. Genotypes as indicated; “-” is no template control. C) Relative quantification of splice variants in wildtype and mutant embryos. Expression levels of each amplicon were quantified and shown normalized to the constant region amplicon. *p = 0.0045.

To explore a role for Elof1 in the regulation of RNApII complex-mediated alternative splicing, we compared the relative abundance of PTB-dependent splice variant at the *Fgfr2* locus. As shown in Fig 4, in *Elof1* mutants we find a significant and specific decrease in the *Fgfr2* splice variant that requires PTB-dependent H3K36me3 (*Fgfr2*-*IIb*). Importantly, we did not observe a relative reduction in the splice isoform that is not PTB-dependent. These data support a hypothesis that *Elof1* is involved in regulation of tissue specific mutually exclusive exon choice during mammalian embryogenesis, and may warrant genome-wide exploration of splice choice in *Elof1* mutant embryos.

## Discussion

Here we show that the transcriptional regulator Elof1 is a critical during early mammalian development. Morphological and histological analyses of Elof1-deficient embryos indicate a failure to initiate gastrulation. These observations are supported by the lack of normal developmental hallmarks and additional defects including failure to initiate T expression, reduced embryonic proliferation and a population of distally positioned but abnormally rounded cells. Together these results demonstrate that murine Elof1, unlike yeast Elf1, is critical for viability and suggests that this highly conserved component of the RNApII elongation complex has acquired additional essential functions throughout evolution.

Although not lethal Elf1 loss of function in yeast results in a phenotypic sensitivity to compounds that deplete nucleotide pools *in vivo* and is consistent with phenotypes produced by loss of other basal components of the RNApII complex [1]. The physical association of yeast Elf1 with the RNApII complex and published phenotypic data suggests that Elf1 plays a role stabilizing the elongation complex and supporting the recruitment of Set2. If mouse Elof1 has a similar role, we hypothesized that loss of Elof1 function could prevent Set2 recruitment resulting in altered H3K36 methylation, which has been shown to correlate with alternative splicing patterns of genes pertinent to development. To better understand why Elof1 loss of function leads to such a dramatic phenotype in the mouse we examined the expression of mutually exclusive splice variants, a form of splicing that is unique to higher eukaryotes. Intriguingly, we document a significant decrease in the abundance of specific PTB-dependent isoforms in Elof1 mutants. In the absence of Elof1 the Fgfr2-IIIB isoform is decreased. Embryos with no functional FgfR2 are peri-implantation lethal, never reaching the egg cylinder stage [30], while loss of the *-IIIb* isoform leads to limb, lung and pituitary defects and lethality at birth [25, 31, 32], indicating critical isoform-specific roles during development. Our data suggests that Elof1 normally influences mutually exclusive splice choice at specific loci with important developmental roles. Although the number of PTB-dependent splice isoforms throughout the mammalian genome is not fully delineated, we hypothesize that the compounding effects of mis-regulation of isoform choice across hundreds or thousands of developmentally important loci could result in the early lethality and gastrulation failure observed in *Elof1* mutants.
